# Construction and Validation of a 15-Top-prognostic-gene-based Signature to Indicate the Dichotomized Clinical Outcome and Response to Targeted Therapy for Bladder Cancer Patients

**DOI:** 10.3389/fcell.2022.725024

**Published:** 2022-03-31

**Authors:** Hongbing Gu, Chaozhao Liang

**Affiliations:** ^1^ Department of Urology, The First Affiliated Hospital of Anhui Medical University, Institute of Urology, Anhui Medical University and Anhui Province Key Laboratory of Genitourinary Diseases, Anhui Medical University, Hefei, China; ^2^ Department of Urology, East District of First Affiliated Hospital of Anhui Medical University, Feidong People’s Hospital, Hefei, China

**Keywords:** bladder cancer, target therapy, signature, prognosis, PDL1

## Abstract

The clinical outcome of heterogeneous bladder cancer (BCa) is impacted by varying molecular characteristics and clinical features, and new molecular classification is necessary to recognize patients with dichotomized prognosis. We enrolled a total of 568 BCa patients from the TCGA-BLCA and GSE13507 cohorts. A total of 107 candidate genes, which were mostly involved in the extracellular matrix-associated pathway, were first selected through the consensus value of the area under the receiver operating characteristic curve (AUC). Furthermore, absolute shrinkage and selection operation regression analysis was implemented to reveal the 15 genes and establish the prognostic signature. The newly defined prognostic signature could precisely separate BCa patients into subgroups with favorable and poor prognosis in the training TCGA-BLCA cohort (*p* < 0.001, HR = 2.41, and 95% CI: 1.76–3.29), as well as the testing GSE13507 cohort (*p* < 0.001, HR = 7.32, and 95% CI: 1.76–3.29) and external validation E-MTAB-4321 cohort (*p* < 0.001, HR = 10.56, 95% CI: 3.208–34.731). Multivariate Cox analysis involving the signature and clinical features indicated that the signature is an independent factor for the prediction of BCa prognosis. We also explored potential targeted therapy for BCa patients with high- or low-risk scores and found that patients with high risk were more suitable for chemotherapy with gemcitabine, doxorubicin, cisplatin, paclitaxel, and vinblastine (all *p* < 0.05), but anti-PD-L1 therapy was useless. We knocked down HEYL with siRNAs in T24 and 5,637 cells, and observed the decreased protein level of HEYL, and inhibited cell viability and cell invasion. In summary, we proposed and validated a 15-top-prognostic gene-based signature to indicate the dichotomized prognosis and response to targeted therapy.

## Introduction

Bladder cancer (BCa), a common disease in the world with an estimated 430,000 new cases diagnosed in 2012, is the ninth most frequent tumor globally. Men have a higher incidence than women, accounting for 75% of patients. European male mortality rates were by far the highest recorded worldwide, especially in Eastern Europe (Antoni et al., 2017). In China, its morbidity and mortality are rising. According to the 2015 National Central Cancer Registry in China, the incidence of BCa ranked sixth in male cancers, with 7.68/10^5^ new cases diagnosed in 2011 (Pang et al., 2016). A total of 3.56/10^5^ persons died of BCa in 2014 (Chen et al., 2018). Among the risk factors, smoking occupies the most important position, and approximately two-thirds of men and one-third of women with BCa are related to smoking (Farling, 2017). There are two types of BCa, muscle invasive BCa (MIBC) and non-muscle invasive BCa (NMIBC). NMIBC is also known as early-stage bladder cancer, and MIBC is an advanced stage with a high recurrence rate (Hautmann et al., 2006; Chamie et al., 2013).

As a heterogeneous disease, the clinical outcome of BCa is impacted by various characteristics in different patients, such as gene mutations, neoantigens, gene copy number alterations, and infiltration of immunocytes. Low-grade tumors have a low progression rate and a low short-term recurrence rate and can be removed easily by transurethral resection (TUR) or intravesical therapy with *Bacillus* Calmette-Guérin (BCG). However, 15.0%–20.0% of NMIBCs ultimately develop into invasive MIBCs (Hedegaard et al., 2016). At the other end of the spectrum, with a high short-term recurrence rate and a high malignant potential leading to tumor progression, high-grade tumors always have a poor prognosis (Kirkali et al., 2005). Therefore, it is important to establish an efficient and concise novel method to discriminate high-risk and low-risk BCa patients. Many teams have attempted to establish a novel classification of BCa at the molecular level. Mo et al. generated an 18-gene signature model to divide MIBC and NMIBC into two subgroups: basal and differentiated. The basal subgroup expressed a low signature gene level, and the differentiated subgroups expressed a high signature gene level. Further study showed a significant difference in overall survival time between the basal and differentiated subgroups. Compared with the differentiated subgroup, the basal subgroup had a worse overall survival outcome (Mo et al., 2018). Sjödahl et al. defined five major urothelial cancer subtypes using 308 cases, and 11 signature genes were identified. They broke the limitation of pathological staging and grading, adding more valuable information for pathological classification (Sjödahl et al., 2012). Kim et al. generated a progression-related gene classifier to predict the disease outcome of NMIBC patients (Kim W. J et al., 2010). Although many prognostic signatures and molecular subtypes have been found, they are still not mature enough to guide clinical therapy such as HER2 for breast cancer (Tsang and Tse, 2020).

Molecular classification is a novel, objectively assessed, individual, and functions as a complement to the classification system. Some genetic events, such as genetic or epigenetic changes that can cause aberrant gene expression, occur in the early stage of BCa (Kim and Quan, 2005; Kim and Bae, 2008). Thus, classification based on gene expression profiling represents a potentially useful way to discriminate different prognosis. In our novel molecular classification module, a total of 1,155 genes from TCGA and GSE13507 cohorts were enrolled and finally generated a 17-gene signature classifier.

## Materials and Methods

### Data Collection

All expression profiles were derived from The Cancer Genome Atlas (TCGA, https://portal.gdc.cancer.gov/) and the Gene Expression Omnibus (GEO, www.ncbi.nlm.nih.gov/gds), and corresponding clinical information was obtained from the TCGA Bladder-cancer Clinical Data Resource dataset and GEO clinical data resource. After matching the available gene expression data and clinical features, a total of 403 patients from the TCGA-BLCA cohort and 165 patients from the GSE13507 cohort were recorded for the utilization of the current study.

### Identification of Candidate Genes

**FIGURE 1:**
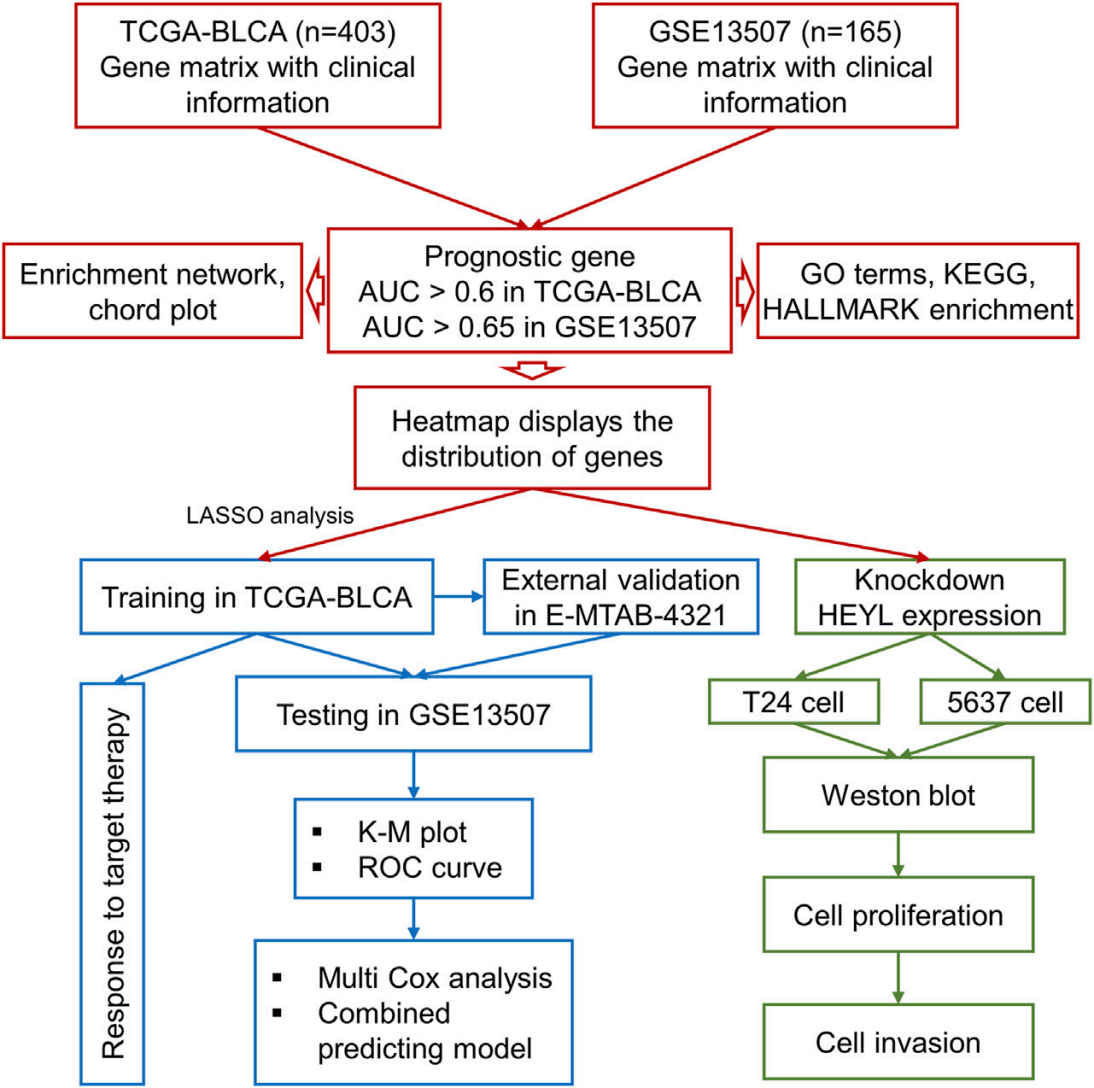
The process of the current study.

Taking the intersection of the expression files of the TCGA-BLCA and GSE13507 cohorts, we finally enrolled 11,255 genes for the subsequent analysis. The receiver operating characteristic (ROC) curve and area under the curve (AUC) were calculated by the “pROC” package to assess the prognostic ability of all included genes and screen the genes included in the analysis with optimal cutoff values (TCGA-BLCA: 0.60; GSE13507: 0.65). Furthermore, to construct an accurate prognostic model, the least absolute shrinkage and selector operation (LASSO) regression analysis was implemented for screening candidate genes by using the “glmnet” package. The minimum lambda value was defined as a cutoff point to minimize the mean cross-validated error. These genes selected *via* LASSO analysis were used to calculate the risk score of each patient with the gene expression value and coefficient. The process of the current study is shown in Figure 1.

### Enrichment Analysis of Selected Genes

The genes with the top mean AUC in both TCGA-BLCA and GSE13507 cohorts were visualized with a heatmap using the R function “barplot”. GO (Gene Ontology), KEGG (Kyoto Encyclopedia of Genes and Genomes), and HALLMARK enrichment analyses of genes were implemented by R package “ClusterProfiler” (Yu et al., 2012) and “msigdbr” (Subramanian et al., 2005). Dot plots of biological processes, molecular function, and cellular components were visualized using the R function “enrichGO”; the connections between biological processes were shown using the R function “emapplot”, the correlation between intersection genes and the top 5 biological process GO terms was demonstrated using the R function “cnetplot”, KEGG pathways were drawn using the R function “enrichKEGG”, and HALLMARK pathways were illustrated using the function “msigdbr” in “clusterProfiler”. Adjusted *p* < 0.05 was set as the cutoff threshold.

### Overall Survival Analysis and Cox Survival Analysis

Kaplan–Meier (K-M) survival analysis and a log-rank test were performed based on the data of candidate gene expression profiles and corresponding clinical parameters to evaluate survival rates by using the “survival” package. The Cox model was established to calculate hazard ratios (HRs) and 95% confidence intervals (CIs). Multivariate Cox regression curves were generated to explore the independent prognostic effect of risk scores after adjusting for several clinical characteristics, including age, sex, and grade. The results were illustrated with multiforest plots. To further explore the impact of the classifier on the prognosis of different subgroups, we conducted a subgroup analysis based on clinical features related to prognosis, including age (≤70 or >70), tumor stage (≤Stage II or >Stage II), sex (male or female), and grade (low or high).

### Prediction of the Response to Targeted Therapy

We predicted the chemotherapeutic response for each sample based on the Genomics of Drug Sensitivity in Cancer (GDSC). Six commonly used chemotherapy drugs, cisplatin, doxorubicin, mitomycin, paclitaxel, vinblastine, and gemcitabine, were selected for assessment. We compared the response to the above six drugs *via* the estimation of the samples’ half-maximal inhibitory concentration (IC_50_) conducted by ridge regression. To evaluate the individual likelihood of responding to immunotherapy, a subclass analysis was performed in response to anti-PD-L1 therapy based on the clinical response of 248 patients with BCa who underwent immunotherapy (Meng, 2021).

### Cell Culture, Proliferation, Invasion, and Western Blotting

We cultured the T24 and 5,637 BCa cell lines in RPMI-1640 medium supplemented with 10% fetal bovine serum at 37°C with 5% CO_2_. The BCa cell lines 5,637 and T24 were respectively transfected with 50 pmol negative control, si-HEYL-1#, and si-HEYL-2# inhibitors *via* Lipofectamine 3,000 (Invitrogen; Thermo Fisher Scientific, Inc.) transfer system. The siRNAs were purchased from Guangzhou Ribobio Co., Ltd., and the sequences are listed in Supplementary Table S1.

We evaluated cell viability *via* the MTT assay. A total of 5,000 cells were seeded into a 24-well plate and cultured for 0, 1, 2, and 3 days. Then, 50 µl of 0.5% MTT reagent was added to each well, and the cells were further cultured for 1.5 h at 37°C and then detected on a microplate reader. The optical density (OD) values were measured at 450 nm.

For cell invasion, we seeded 20,00 cells in the upper Transwell chamber (8 μm; Corning, Inc.); Matrigel at a 1:20 concentration was precoated and cultured at 37°C for 2 h. The lower chamber was filled with complete medium as a chemoattractant. After culturing the coculture system at 37°C for 24 h, the chamber and cells were mixed with formalin for 15 min and further stained with 1% crystal violet. The invaded cells were counted in 3 repeated groups (magnification, ×200) under a light microscope (Nikon Corporation).

Western blotting was performed to confirm the knockdown of HEYL at the protein level. Anti-HEYL and anti-tubulin antibodies were used to detect the protein lanes.

### Statistical Analysis

All analyses were completed by R software v4.0.3 (http://www.r-project.org). The risk score of each BCa patient was calculated with the sum of the selected 15 gene coefficients by LASSO regression. The high-risk group and low-risk group were discriminated by the median value of the risk score. Heatmap and enrichment analyses were applied to the visualized expression files. K-M survival analysis was performed to explore the survival difference between the high-risk and low-risk score groups. A log-rank test was used to estimate the survival analysis. HR and 95% CI were calculated by the Cox model. The independent prognostic effect of the risk score was calculated by multivariate Cox regression analysis. *p* < 0.05 was considered a statistically significant difference.

## Result

### Gene Selection

**FIGURE 2:**
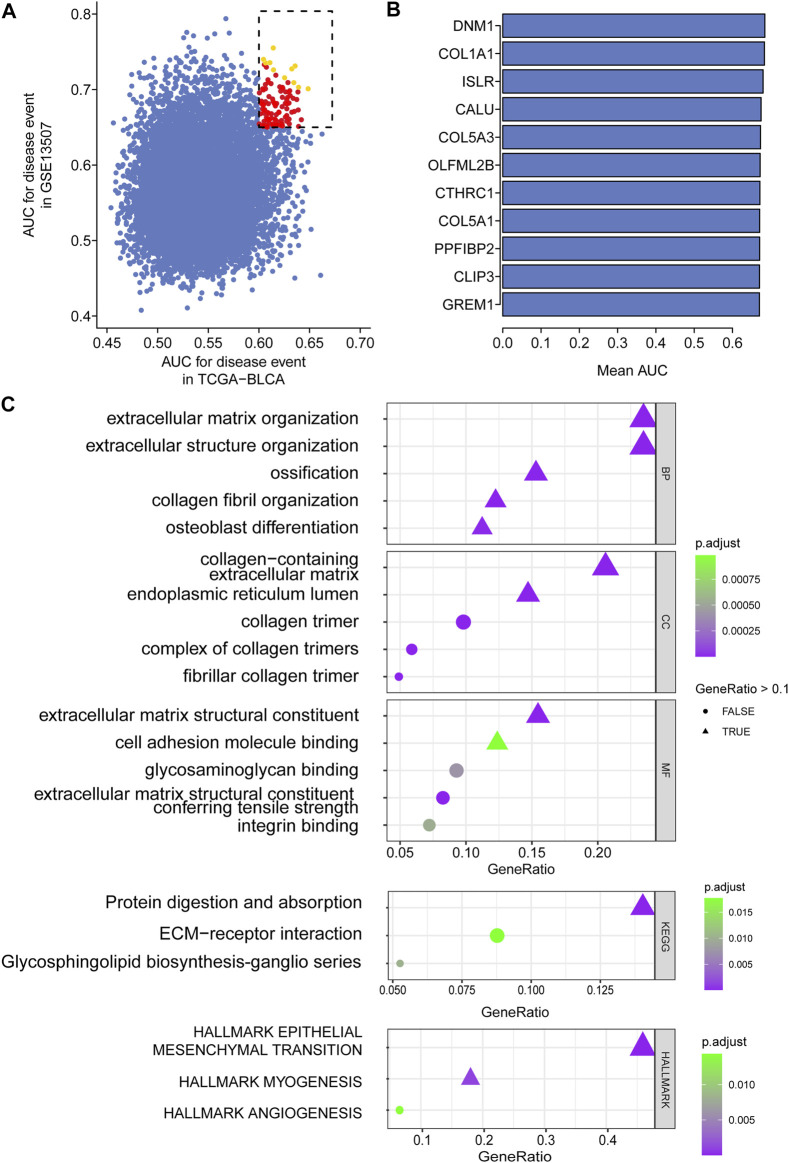
Top prognostic gene selection in both TCGA-BLCA and GSE13507 cohorts. **(A)** AUC evaluation of the prognostic value of candidate genes involved in overall survival based on datasets TCGA and GSE10816. **(B)** The average AUC value of the top 11 candidate genes. **(C)** Annotation of enriched signaling pathways by GO, KEGG, and HALLMARK.

In total, 403 patients with 14,163 genes in TCGA-BLCA and 165 patients with 18,561 genes in GSE13507 were first enrolled, and the corresponding clinical information was also downloaded. Following preprocessing of raw data, 11,255 intersecting genes were subjected to the calculation of AUC to estimate the prediction of the prognosis of BCa patients in those two cohorts (Figure 2A). Genes with the top mean AUC are shown in Figure 2B, including “GREM1”, “CLIP3”, “PPFIBP2”, “COL5A1”, “CTHRC1”, “OLFML2B″, “COL5A3”, “CALU”, “ISLR”, “COL1A1”, and “DNM1”. With the preset cutoff value of AUC (TCGA-BLCA: 0.60; GSE13507: 0.65), we finally enrolled 107 genes for the subsequent analysis.

### Intersection Gene Enrichment and Annotation

**FIGURE 3:**
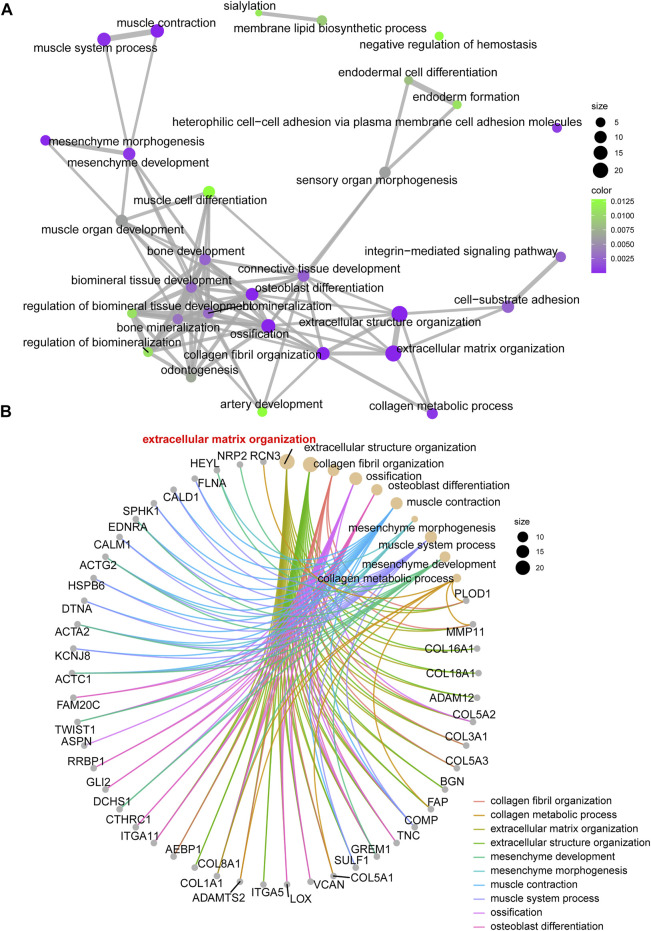
The network and chord graph shows the most important pathways. **(A)** Network of enriched signaling pathways; **(B)** Chord graph of the enriched signaling pathways.

For the enrolled 107 candidate genes, we performed enrichment analysis to reveal their potential function in BCa. The results demonstrated the top five biological process, cellular component, and molecular function GO terms, KEGG terms, and HALLMARK terms (Figure 2C). We concluded that the extracellular matrix structural process, cell adhesion molecule binding, ECM–receptor interaction, and hallmark epithelial–mesenchymal transition signaling pathways play pivotal roles in the tumorigenesis of BCa. The details of the enriched pathways and genes are displayed in Table 1. The interrelation of the top 30 enriched biological processes is presented in Figure 3A, and the correlation between intersected genes and extracellular matrix-associated signaling is also illustrated in Figure 3B.

**TABLE 1:** The details of the enriched signaling pathways of the 107 prognostic genes.

ONTOLOGY BP				
Terms	*p*-value	*p*.adjust	*q*-value	geneID
Extracellular matrix organization	1.16E-18	1.26E-15	1.06E-15	PLOD1/MMP11/COL16A1/COL18A1/ADAM12/COL5A2/COL3A1/COL5A3/BGN/FAP/COMP/TNC/GREM1/SULF1/COL5A1/VCAN/LOX/ITGA5/ADAMTS2/COL1A1/COL8A1/AEBP1/ITGA11
Extracellular structure organization	1.23E-18	1.26E-15	1.06E-15	PLOD1/MMP11/COL16A1/COL18A1/ADAM12/COL5A2/COL3A1/COL5A3/BGN/FAP/COMP/TNC/GREM1/SULF1/COL5A1/VCAN/LOX/ITGA5/ADAMTS2/COL1A1/COL8A1/AEBP1/ITGA11
Collagen fibril organization	6.24E-17	4.27E-14	3.59E-14	PLOD1/MMP11/COL5A2/COL3A1/COL5A3/COMP/GREM1/COL5A1/LOX/ADAMTS2/COL1A1/AEBP1
Ossification	2.40E-09	1.23E-06	1.03E-06	CTHRC1/DCHS1/GLI2/COL5A2/COMP/TNC/GREM1/VCAN/LOX/COL1A1/RRBP1/ASPN/TWIST1/ITGA11/FAM20C
Osteoblast differentiation	2.73E-08	1.12E-05	9.41E-06	CTHRC1/GLI2/TNC/GREM1/VCAN/LOX/COL1A1/RRBP1/TWIST1/ITGA11/FAM20C
ONTOLOGY CC				
collagen-containing extracellular matrix	1.63E-15	3.32E-13	2.86E-13	CTHRC1/COL16A1/COL18A1/COL5A2/COL3A1/COL5A3/BGN/COMP/TNC/GREM1/SULF1/COL5A1/VCAN/NCAM1/ADAMTS2/CDH2/COL1A1/COL8A1/AEBP1/ASPN/SERPINE2
collagen trimer	2.52E-11	2.57E-09	2.21E-09	CTHRC1/COL16A1/COL18A1/COL5A2/COL3A1/COL5A3/COL5A1/LOX/COL1A1/COL8A1
endoplasmic reticulum lumen	6.16E-11	4.19E-09	3.61E-09	RCN3/COL16A1/COL18A1/COL5A2/COL3A1/COL5A3/TNC/COL5A1/VCAN/PDIA5/CDH2/COL1A1/COL8A1/FAM20C/CALU
complex of collagen trimers	4.24E-10	2.16E-08	1.86E-08	COL5A2/COL3A1/COL5A3/COL5A1/COL1A1/COL8A1
fibrillar collagen trimer	1.51E-09	5.14E-08	4.43E-08	COL5A2/COL3A1/COL5A3/COL5A1/COL1A1
ONTOLOGY MF				
extracellular matrix structural constituent	1.22E-14	3.35E-12	3.03E-12	CTHRC1/COL16A1/COL18A1/COL5A2/COL3A1/COL5A3/BGN/COMP/TNC/COL5A1/VCAN/COL1A1/COL8A1/AEBP1/ASPN
extracellular matrix structural constituent conferring tensile strength	5.00E-11	6.85E-09	6.19E-09	COL16A1/COL18A1/COL5A2/COL3A1/COL5A3/COL5A1/COL1A1/COL8A1
glycosaminoglycan binding	4.63E-06	0.000423	0.000382	TNFAIP6/COL5A3/BGN/COMP/NRP2/SULF1/COL5A1/VCAN/SERPINE2
integrin binding	8.05E-06	0.000552	0.000498	COL16A1/COL3A1/FAP/COMP/THY1/COL5A1/ITGA5
cell adhesion molecule binding	1.79E-05	0.000983	0.000887	PVR/COL16A1/COL3A1/FAP/COMP/THY1/COL5A1/ITGA5/CALD1/CDH2/CNN3/FLNA
KEGG				
Protein digestion and absorption	5.22E-07	7.57E-05	6.65E-05	COL16A1/COL18A1/COL1A1/COL3A1/COL5A1/COL5A2/COL5A3/COL8A1
Glycosphingolipid biosynthesis—ganglio series	0.000143	0.010337	0.00908	B4GALNT1/ST3GAL5/ST6GALNAC5
ECM–receptor interaction	0.000366	0.017704	0.015551	COL1A1/COMP/ITGA11/ITGA5/TNC
HALLMARK				
HALLMARK_EPITHELIAL_MESENCHYMAL_TRANSITION	2.29E-22	6.86E-21	6.26E-21	ACTA2/ADAM12/BGN/CALD1/CALU/CDH2/COL16A1/COL1A1/COL3A1/COL5A1/COL5A2/COL5A3/COMP/CTHRC1/FAP/FLNA/GEM/GREM1/ITGA5/LOX/PLOD1/PRRX1/PVR/SERPINE2/TAGLN/THY1/TNC/VCAN
HALLMARK_MYOGENESIS	7.61E-05	0.001142	0.001041	ACTC1/ADAM12/AEBP1/CNN3/COL1A1/COL3A1/DTNA/MAPK12/NCAM1/SPHK1/TAGLN
HALLMARK_ANGIOGENESIS	0.001434	0.01434	0.013082	COL3A1/COL5A2/KCNJ8/VCAN

### Construction of the Prognostic Signature

**FIGURE 4:**
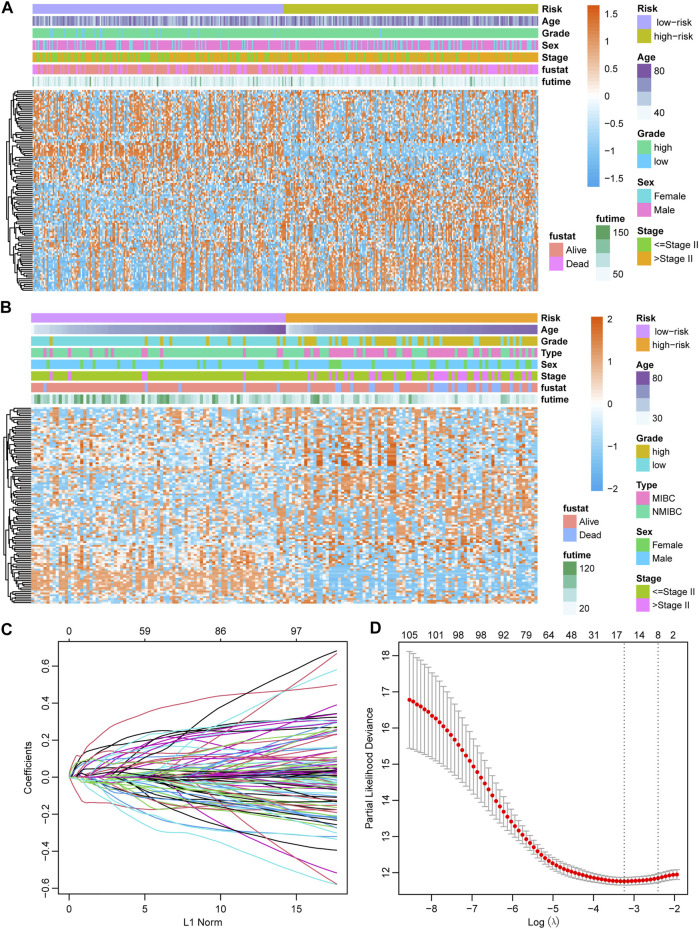
Establishment of the prognostic model **(A)** Heatmap showing the expression of 107 candidate genes in the TCGA-BLCA cohort. **(B)** Heatmap showing the expression of 107 candidate genes in the GSE13507 cohort. **(C)** The optimal tuning parameter (lambda) in the LASSO analysis selected with 5-fold cross-validation and one standard error rule. **(D)** LASSO coefficient profiles of the 107 candidate genes.

The gene expression of 107 genes and associated clinical features for the TCGA-BLCA cohort are shown in Figure 4A, and those for the GSE13507 cohort are shown in Figure 4B. LASSO regression was performed to identify candidate genes and evaluate the corresponding coefficients in the TCGA-BLCA training cohort (Figures 4C,D). Based on the minimum lambda value of 0.032, a total of 15 genes were enrolled for the calculation of the prognostic signature, with the formula risk score = 0.109 × expression of PHGDH − 0.117 × expression of CD96 + 0.007 × expression of SETBP1 + 0.077 × expression of GALK1 + 0.019 × expression of DTNA +0.064 × expression of SERPINB2 + 0.018 × expression of COMP +0.043 × expression of CALM1 + 0.111 × expression of HEYL +0.022 × expression of CCRN4L + 0.032 × expression of FADS2 + 0.083 × expression of TMEM109 – 0.013 × expression of CTSE +0.021 × expression of FAM43A − 0.022 × expression of IL9R. The risk score for each patient in both TCGA-BLCA and GSE13507 cohorts was calculated along with the above-mentioned formula. For the subsequent analysis, the prognostic value of each single gene is displayed in Supplementary Figure S1.

### Prognostic Value of the Newly Defined Signature

**FIGURE 5:**
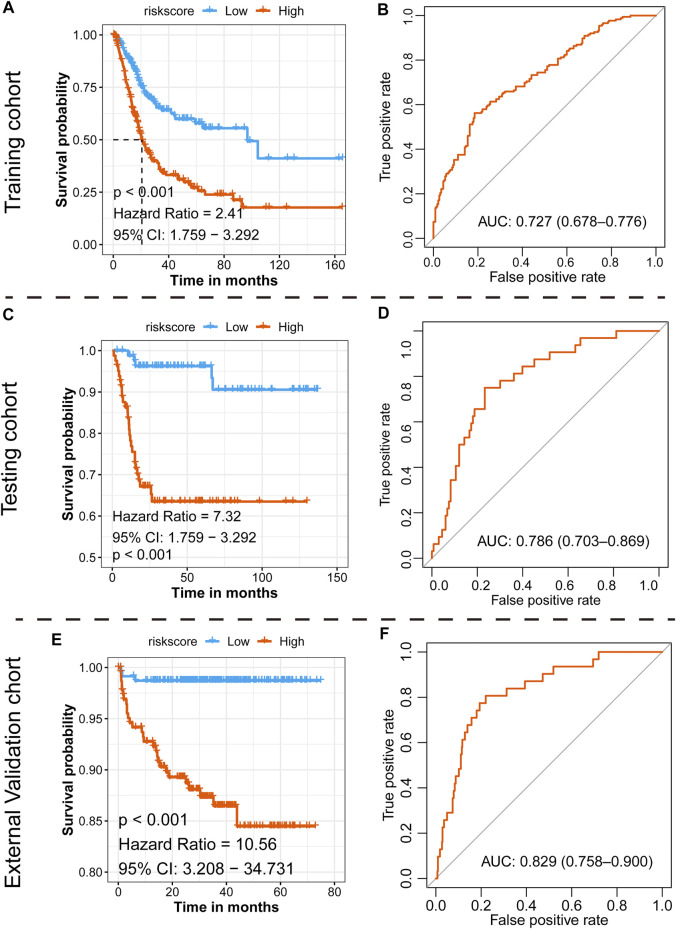
Prognostic value of the newly defined signature. **(A)** K-M plot showing the separated clinical outcome of patients belonging to the high-risk and low-risk subgroups in the training TCGA-BLCA cohort **(A)**, testing GSE13507 cohort **(C)**, and external validation cohort **(E)**; ROC curve showing the prognostic value of the signature in the training TCGA-BLCA cohort **(B)**, testing GSE13507 cohort **(D)**, and external validation cohort **(F)**.

With the median risk score as the cutoff point, patients in the TCGA-BLCA cohort were divided into the high-risk class (*n* = 201) and low-risk class (*n* = 198) (Supplementary Figure S2A), and the results of the risk map showed a significant survival difference between the two classes (Supplementary Figure S2A). K-M curves demonstrated that the low-risk class had a better overall survival time than the high-risk class (*p* < 0.001, HR = 2.41, 95% CI: 1.76–3.29) (Figure 5A), and the ROC curve showed that the AUC of this classification strategy could be 0.727, with a 95% CI of 0.678–0.776, which indicated an outstanding prognostic value (Figure 5B). The distribution of the clinical features in the high-risk and low-risk groups of TCGA-BLCA are listed in Table 2. We also assessed the prognostic value in different subgroups of patients. The signature was meaningful for BCa patients in the subgroups of age ≤ 70 years old (*p* < 0.01), age >70 years old (*p* < 0.01), Stage I + II (*p* = 0.02), Stage III + IV (*p* < 0.01), male sex (*p* < 0.01), and high grade (*p* < 0.01) in the TCGA-BLCA cohort (Supplementary Figure S3).

**TABLE 2:** Summary of clinical features in the TCGA-BLCA cohort.

	Level	Low risk	High risk	*p*
Survival time		29.43 ± 28.66	23.79 ± 25.37	0.038
Survival status (%)	0	140 (70.7)	84 (41.8)	<0.001
	1	58 (29.3)	117 (58.2)	
Stage (%)	≤Stage II	84 (42.4)	45 (22.4)	<0.001
	>Stage II	114 (57.6)	156 (77.6)	
Sex (%)	Female	44 (22.2)	61 (30.3)	0.084
	Male	154 (77.8)	140 (69.7)	
Grade (%)	High	179 (90.4)	200 (99.5)	<0.001
	Low	19 (9.6)	1 (0.5)	
Age		66.62 ± 10.64	69.08 ± 10.24	0.019
Signature		1.92 ± 0.33	2.69 ± 0.26	<0.001

### Assessing the Prognostic Value of the Signature in the Testing GSE13507 Cohort

To validate the 15-gene signature predictive values in other BCa cohorts, the same formula was conducted in the GSE13507 dataset to generate the risk score of each patient. Similarly, patients were divided into a high-risk group and a low-risk group based on the median risk score as the cutoff point (Supplementary Figure S2B). Consistent with the results in the TCGA-BLCA cohort, the low-risk group exhibited a shorter overall survival time than the high-risk group (*p* < 0.001, HR = 7.32, and 95% CI: 1.76–3.29, Figure 5C), and the AUC was 0.786, with a 95% CI of 0.703–0.869 (Figure 5D). The distribution of the clinical features in the high-risk and low-risk groups of GSE13507 is listed in Table 3. We also assessed the prognostic value in different subgroups of patients. The signature was meaningful for BCa patients in the subgroups of age ≤ 70 years old (*p* < 0.01), age >70 years old (*p* < 0.01), Stage I + II (*p* = 0.027), male (*p* < 0.01), female (*p* < 0.01), low grade (*p* = 0.018), and high grade (*p* < 0.01) in the GSE13507 cohort (Supplementary Figure S4).

**TABLE 3:** Summary of clinical features in the GSE13507 cohort.

	Level	Low risk	High risk	*p*
Survival time		55.60 ± 37.87	29.70 ± 30.42	<0.001
Survival status (%)	0	108 (90.8)	25 (54.3)	<0.001
	1	11 (9.2)	21 (45.7)	
Stage (%)	≤Stage II	106 (89.1)	23 (50.0)	<0.001
	>Stage II	13 (10.9)	23 (50.0)	
Sex (%)	Female	17 (14.3)	13 (28.3)	0.063
	Male	102 (85.7)	33 (71.7)	
Type (%)	MIBC	25 (21.0)	37 (80.4)	<0.001
	NMIBC	94 (79.0)	9 (19.6)	
Grade (%)	High	27 (22.7)	33 (71.7)	<0.001
	Low	92 (77.3)	13 (28.3)	
Age		64.59 ± 12.65	66.72 ± 9.98	0.258
Signature		4.57 ± 0.26	5.26 ± 0.19	<0.001

### Assessing the Prognostic Value of the Signature in the External Validation E-MTAB-4321 Cohort

To validate the 15-gene signature predictive values in other BCa cohorts, the same formula was conducted in the E-MTAB-4321 dataset to generate the risk score of each patient. Similarly, patients were divided into a high-risk group and a low-risk group based on the median risk score as the cutoff point (Supplementary Figure S2C). Consistent with the results in the TCGA-BLCA cohort, the low-risk group exhibited a shorter overall survival time than the high-risk group (*p* < 0.001, HR = 10.56, and 95% CI: 3.21–34.73, Figure 5E), and the AUC was 0.829, with a 95% CI of 0.758–0.900 (Figure 5F). We also assessed the prognostic value in different subgroups of patients. The signature was meaningful for BCa patients in the subgroups of age < = 70 years old (*p* = 0.004), age >70 years old (*p* < 0.001), stage CIS and Ta (*p* = 0.004), stage T1–T4 (*p* = 0.019), male sex (*p* < 0.001), low grade (*p* < 0.001), and high grade (*p* = 0.045) in the E-MTAB-4321 cohort (Supplementary Figure S5).

### Independent Prognostic Value of the Risk Score

**FIGURE 6:**
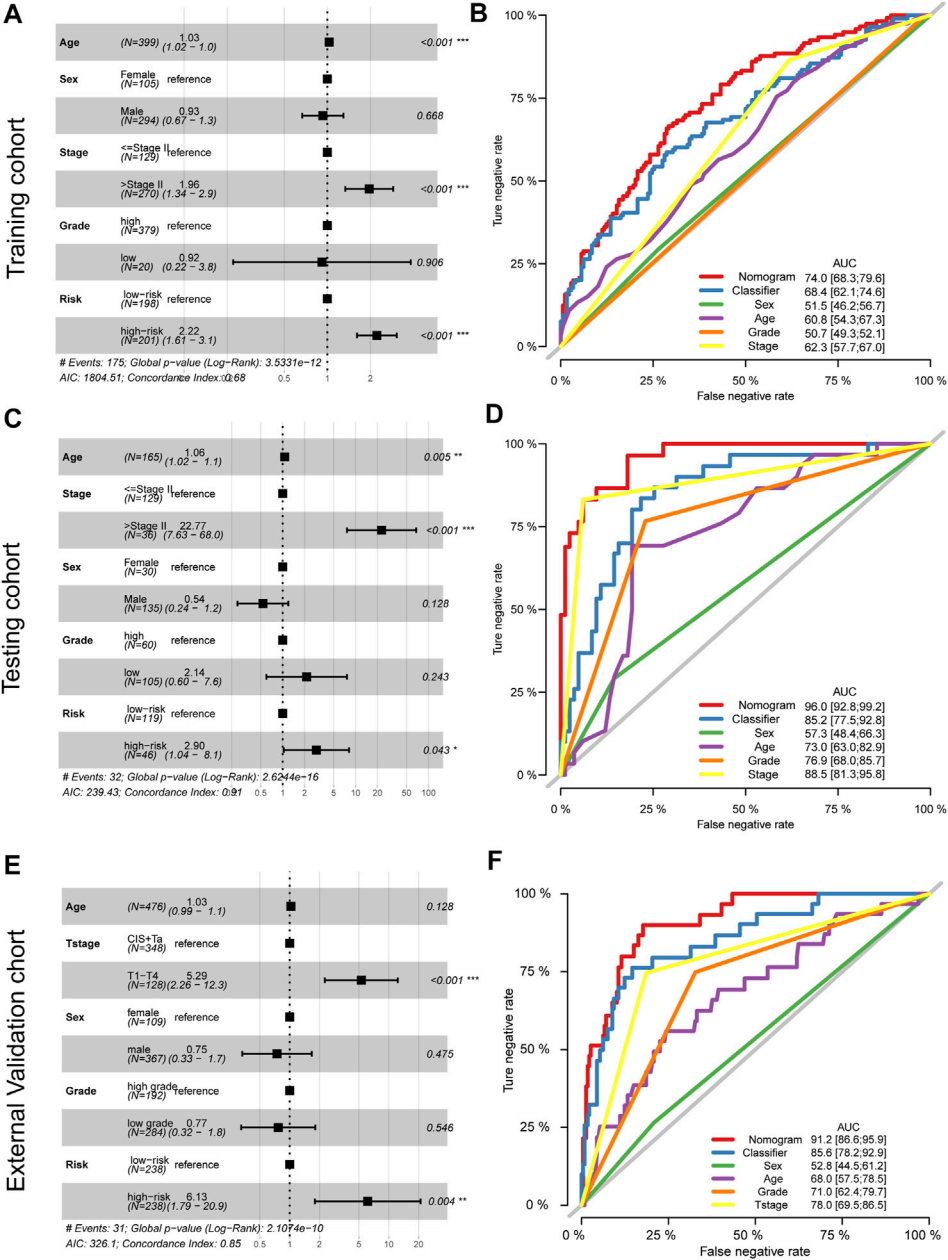
Adjustment and combination of the clinical features with the prognostic signature. Multivariate Cox analysis and forest plot revealed the independent prognostic value of the signature in the training TCGA-BLCA cohort **(A)**, testing GSE13507 cohort **(C)**, and external validation cohort **(E)**. The combination nomogram of the signature and clinical features displays a preferred prognostic value in the training TCGA-BLCA cohort **(B)**, testing GSE13507 cohort **(D)**, and external validation cohort **(F)**.

To further explore the signature usage, multivariate Cox regression analysis was performed in the TCGA-BLCA cohort, and as the results showed, age, stage, and risk score were independent prognostic factors (*p* < 0.001). Even after adjusting for the impact of other clinical features, the signature defined high-risk patients as having an approximately 2.22-fold higher risk of death than low-risk patients (Figure 6A). A combined nomogram enrolling both the signature classifier and other clinical features demonstrated a better prognostic value (AUC: 0.740, 95% CI: 0.683–0.796, Figure 6B). For the GSE13507 cohort, we also revealed similar findings. The prognostic signatures defined as high-risk patients had a 2.90-fold higher risk of death than low-risk patients, which acted as an independent prognostic factor after adjusting for the features of age, tumor stage, sex, and grade (Figure 6C). Moreover, the combined nomogram illustrated a prognostic AUC value as high as 0.960, with a 95% CI of 0.928–0.992 (Figure 6D). For the E-MTAB-4321 cohort, the prognostic signatures defined as high-risk patients had a 6.13-fold risk of BCa recurrence compared with low-risk patients, which acted as an independent prognostic factor after adjusting for the features of age, T stage, sex, and grade (Figure 6E). The combined nomogram illustrated a prognostic AUC value as high as 0.912, with a 95% CI of 0.866–0.959 (Figure 6F).

### Exploring the Appropriate Targeted Therapy for BCa Patients

**FIGURE 7:**
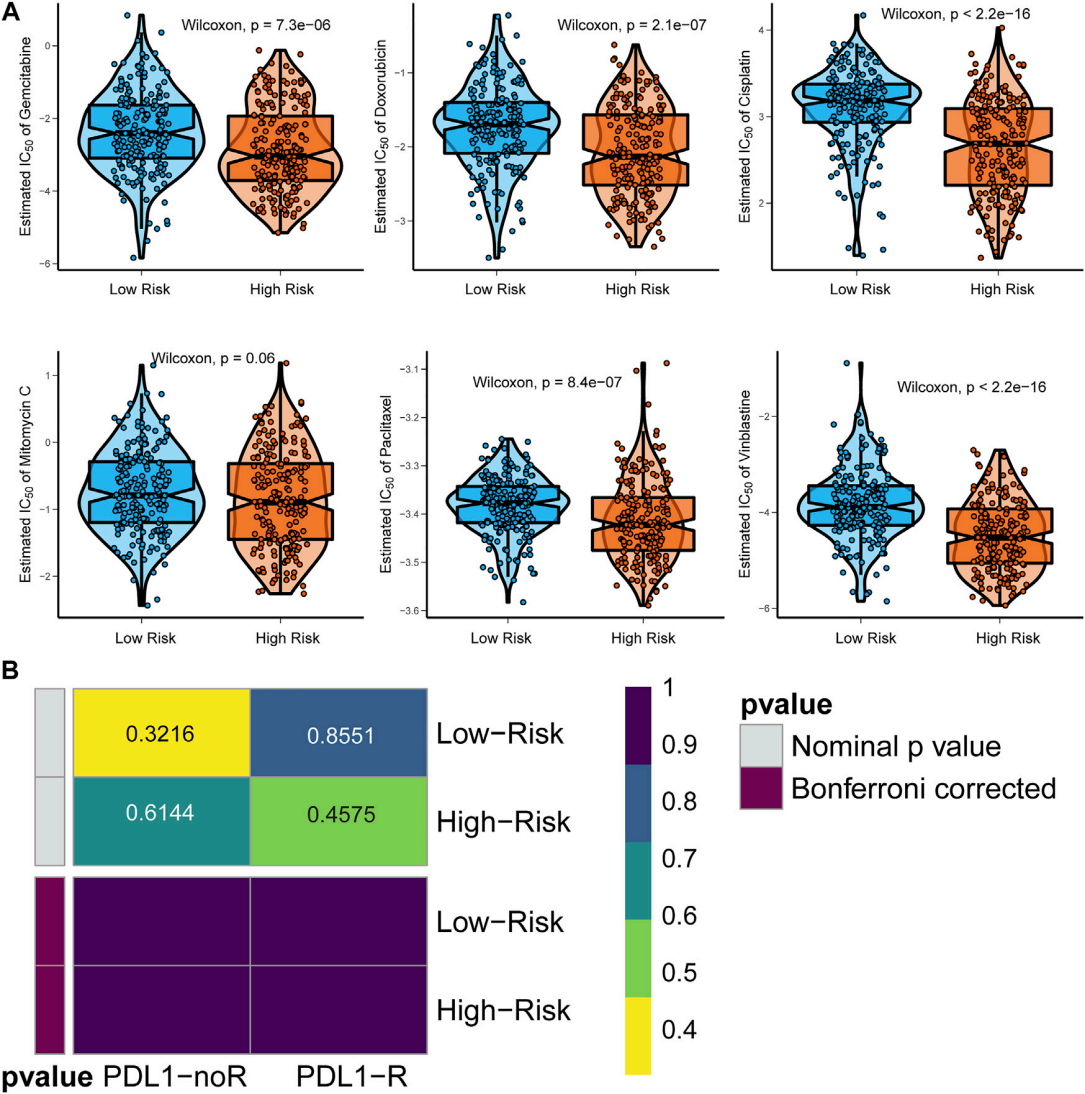
Identification of appropriate targeted therapy for BCa patients. **(A)** Appropriate chemotherapy for signature-defined high-risk and low-risk patients. **(B)** Appropriate anti-PD-L1 immunotherapy for signature-defined high-risk and low-risk patients.

Six chemotherapy drugs, cisplatin, doxorubicin, mitomycin, paclitaxel, vinblastine, and gemcitabine, are commonly used for the clinical treatment of BCa. We compared the response of the six drugs by assessing the IC50 after comparison with the GDSC data. We found that patients in the high-risk group were more suitable for treatment with gemcitabine, doxorubicin, cisplatin, paclitaxel, and vinblastine (all *p* < 0.05), but not mitomycin (*p* = 0.06, Figure 7A). For immunotherapy, especially anti-PD-L1 therapy, we employed the gene expression data of the IMvigor210 cohort. After comparing the similarity of the gene expression profile of responders with the patients in both the high-risk and low-risk groups, we found that both patients in the two subgroups were not suitable for treatment with anti-PD-L1 therapy (Figure 7B).

### Knockdown of HEYL Inhibited the Proliferation and Invasion of BCa Cells

**FIGURE 8:**
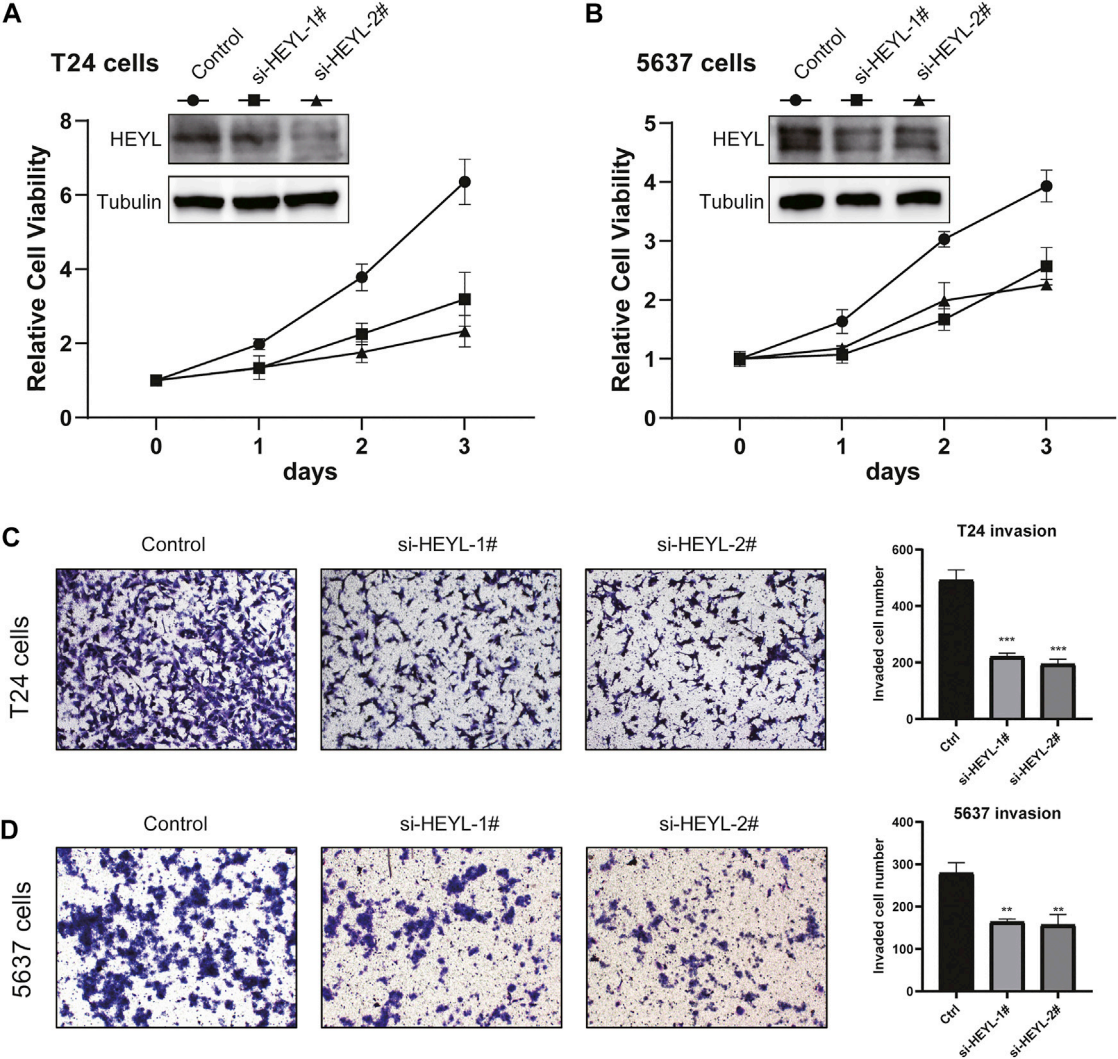
Phenotypic validation of HEYL knockdown in BCa cell lines. **(A)** Knockdown of HEYL inhibited the viability of T24 cells. **(B)** Knockdown of HEYL inhibited the viability of 5,637 cells. **(C)** Knockdown of HEYL inhibited the invasion of T24 cells. **(D)** Knockdown of HEYL inhibited the invasion of 5,637 cells.

To evaluate the functions of the newly defined signature, we evaluated the phenotypic alterations of BCa cells after knockdown HEYL, due to the fact that HEYL was given a large weight of 0.111 in the risk score formula. We transferred the control, si-HEYL-1#, and si-HEYL-2# by the Lipofectamine 3,000 system to both T24 and 5,637 cells and observed decreased protein levels of HEYL (Figures 8A,B **)**. Cell viability was also inhibited after knockdown by two siRNAs (Figures 8A,B). For cell invasion, which represents tumor malignancy, we observed similar results: the invaded cell numbers significantly decreased in the si-HEYL-1# (all *p* < 0.001, Figure 8C) and si-HEL-2# (all *p* < 0.01, Figure 8D) groups compared with the control group in both T24 and 5,637 cells.

## Discussion

BCa ranks as the fourth most common malignant cancer among men in the Western world (Kirkali et al., 2005). BCa is a type of tumor with a strong correlation between age and sex. The median age at diagnosis is approximately 65–70 years old. The incidence rate in men is 3–4 times that in women (Chen et al., 2018). However, according to the Global Cancer Incidence and Mortality Survey, the age-standardized rates (ASRs per 100,000) of women are 5.7, and the ASRs of men are 9.6, suggesting that the stage-adjusted survival of BCa in women is poorer than that in men (Ferlay et al., 2019). There is a detectable difference among variant races and regions or countries. For instance, white Americans are more susceptible to BCa than African Americans. On the other hand, white Americans are more likely to evolve into invasive tumors and have a higher mortality rate than African Americans (Uno et al., 2000). With the development of sequencing and complementary technologies, molecular classification methods based on genes have been increasingly studied. Compared to the traditional pathology-based classification, a novel subtyping strategy involves more tumor biological information and is expected to have broad application prospects.

Great progress has been made in the study of gene expression profiles and cancer prognosis. For instance, patients with HER2-enriched breast cancer have poor clinical outcomes, but they are sensitive to neoadjuvant chemotherapy, which can greatly benefit this group (Ross et al., 2003). However, only limited data are available for predicting BCa and prognosis to date. Different teams generate a distinct subtyping system, and each has its own characteristics and validity. Either they were created to cater to a specific clinical therapy project or relied on a robust statistical method without considering the cancer biological process at data analysis (Zhu et al., 2020). In our study, we downloaded two data sets from different sources, and a total of 107 prognostic genes were identified for LASSO analysis. To further explore the functions of the 107 candidate genes, GO enrichment analysis was performed. As the results showed, the candidate gene expression was mainly involved in five pathways, including extracellular matrix organization, extracellular structure organization, ossification, collagen fibril organization, and osteoblast differentiation. Moreover, we established the prognostic signature by LASSO analysis of selected genes and generated a risk predictive model with one of them. The formula risk score = $\;\;\overset{n}{\underset{i\;=\;1}{\sum }}\alpha *Xi\;$ (αrepresent coef. min) was used to calculate the risk score of each patient. Selecting an optimal cutoff point, the high-risk group and low-risk group were separated. With a robust statistical method, we found that patients in the high-risk group exhibited a worse prognosis than those in the low-risk group. Independently, the same formula and statistical strategy were performed on the testing GSE13507 cohort and external validation E-MTAB-4321 cohort to assess the efficacy of the model, and the result was similar to that in the TCGA-BLCA cohort. Furthermore, we also realized that the newly defined signature is an independent prognostic signature after adjusting for the clinical features of age, tumor stage, sex, and grade.

For the 15 genes enrolled for the calculation of the prognostic signature, several basic experiments have already demonstrated their function in the tumorigenesis of BCa. DNTA plays a role in cell signal transduction and mediates the Notch1 pathway axis. This pathway was reported to be an essential regulator in cell proliferation, differentiation, and apoptosis (Kim M. Y et al., 2010), and the Notch1 pathway has also been proven to contribute to the metastasis of various malignancies, including ovarian, breast, lung, and renal cancer (Kong et al., 2016). HEYL is the target gene of the Notch1 pathway, and there is limited information on the impact of its target gene HEYL. The results of Weber et al. showed that an enhanced expression level of HEYL decreased cancer cell dissemination and the absolute number of metastases formed, while the capacity of cell metastasis remained good, indicating that HEYL can function as a negative regulator by inhibiting the infiltration of metastasis-initiating cells (Weber et al., 2019). At the same time, we found two other tumor suppression genes, SETBP1 and SULF1 (Lai et al., 2008; Li et al., 2020). It was reported that decreased expression of SETBP1 contributed to the development of non-small-cell lung cancer cells by increasing tumor cell proliferation, migration, and invasion and was associated with poor prognosis. In addition, other studies have claimed that SETBP1 expression is associated with acute myeloid leukemia and that microRNA-211-5p directly targets SETBP1 to inhibit triple-negative breast cancer cell proliferation, migration and metastasis (Chen et al., 2017). SULF1 is increasingly considered for its tumor suppressor effect. The target pathways related to the effect of SULF1 include hedgehog, Wnt, and multiple heparan sulfate-dependent receptor tyrosine kinase pathways, which may prove to be an important method to prevent and treat cancer (Lai et al., 2008).

We also revealed six oncogenes that have been validated to be related to the poor prognosis of patients with cancer: CTSE, CALM1, PHGDH, IL9R, and CD96 (Boulay et al., 2001; Possemato et al., 2011; Yan et al., 2011; Pontious et al., 2019; Salazar et al., 2020; Zhao et al., 2020; Liu et al., 2021). CTSE is an intracellular hydrolytic aspartic protease that has been found to be overexpressed in cancer tissues. With the help of endoscopy and immunosorbent assay (ELISA) and Western blot, CTSE was considered a better biomarker than CA19-9 for detecting pancreatic cancer. CALM1, a calcium ion (Ca2+) receptor protein, is responsible for mediating various signaling processes. Overexpression of CALM1 in cancer is significantly related to clinical stage, T classification, and poor prognosis. The function of CALM1 depends on the synergy of ERGF, and similar to anti-EGFR antibodies, CALM1 inhibitors play an essential role in cancer chemotherapy (Liu et al., 2021). PHGDH is a key enzyme in the serine synthesis pathway. Serine is an intermediate of other amino acids and lipid and nucleic acid synthesis pathways (Locasale et al., 2011) and thus promotes cancer progression. Studies have shown that PHGDH is a reliable biomarker and independent factor predicting prognosis (Song et al., 2018). IL9 is a multifunctional cytokine involved in many pathways in the cell and plays opposite roles in different tumors. For instance, IL9 inhibited the proliferation of the gastric cancer cell line SGC-7901 *in vitro* (Cai et al., 2019) through the activation of adaptive or innate immune responses. However, IL9 can act as a tumorigenic factor or an enhancing factor to promote the proliferation of hematological tumors and some solid tumors (Chen and Wang, 2014; Hu et al., 2017). CD96 is mainly involved in immune function, especially the immune response mediated by T cells. CD96 can promote cancer metastasis by enhancing NK cell-target adhesion and inhibiting the NK-mediated cytokine response (Liu et al., 2020). Interestingly, high expression of CD96 inhibits IL9 production in Th9 cells (Stanko et al., 2018).

The expression level of SERPINB2 was consistent with carcinogen exposure, indicating that SEPRINB2 may provide sensitive and accurate information for the beginning of tumorigenic events (Lee et al., 2019). SERPINB2 was also reported as a regulator or biomarker for predicting the malignant progression of colorectal and bladder cancer (Ganesh et al., 1994; Champelovier et al., 2002). Importantly, an increase in SERPINB2 was detected with most of the additional tested tumorigenic substances, which adds more evidence for SEPRINB2 as a potential biomarker. Liu et al. found that COMP was an excellent prognostic factor and biomarker of colon cancer equivalent to noninvasive biomarker performance, such as CA-199 (Liu et al., 2018). Studies have shown that COMP contributes to the development and metastasis of breast cancer. The enhanced expression level of COMP in tumor cells is significantly related to the reduced breast cancer-specific survival rate and recurrence-free survival rate of patients, while the expression level of COMP in the stroma has a poor connection with prognosis (Englund et al., 2016). CCRN4L, a type of clock-control gene, is a key component in the regulation of circadian rhythms (Filipski and Lévi, 2009). It was considered that the expression of clock-control genes changed in some cancer groups, and polymorphisms in the CCRN4L gene may contribute to the genesis of NSCLC in Brazilian patients (Couto et al., 2014). FADS2 is a potential oncogene (Tian et al., 2020). Several cancers have been validated to utilize FADS2 to desaturate palmitate to the unusual fatty acid sapienate, which can be applied to the biosynthesis of the membrane. Sapienate biosynthesis is an alternative method for fatty acid desaturation metabolism in cancer cells (Vriens et al., 2019).

As we know, this is the first study that concerned the top prognosis prediction genes to construct the signature; we selected the top prognostic genes with the high AUC values in both TCGA-BLCA and GSE13507 cohorts, and the highly prognostic efficacy of the 15-gene signature was displayed in both TCGA-BLCA and GSE13507 cohorts, as well as the external validation E-MTAB-4321 cohort. Meanwhile, several limitations of the current study should be illustrated. First, real-world patient cohort is needed to further validate the prognostic value of the 15-gene signature. Second, experimental studies of the enrolled 15 genes are necessary to further reveal the potential mechanisms of them in BCa tumorigenesis. Third, the strength of evidence for the significance of therapy is not enough, and further mechanism study and clinical experiment should be conducted to evaluate the targeted therapy efficacy.

## Conclusion

As gene mutation events accumulate, tumor cells gradually develop. Molecular changes occur earlier than clinical symptom onset and overt radiographic evidence. Therefore, molecular classifiers are considered promising tools for the early prediction of tumors. BCa is a disease with multiple factors and heterogeneity, and traditional classification cannot reflect the actual situation and prognosis. In the current study, we enrolled 15 genes that can delineate BCa from multiple angles, and the 15-gene classifier was validated to be associated with the clinical outcome and response to targeted therapy of patients with BCa.

## Data Availability

The datasets presented in this study can be found in online repositories. The names of the repository/repositories and accession number(s) can be found in the article/Supplementary Material.

## Abbreviations

BCa, Bladder cancer; DEG, Differentially expressed gene; GEO, Gene Expression Omnibus; KM, Kaplan–Meier; ROC, Receiver operating characteristic; TCGA, The Cancer Genome Atlas.

## References

[B1] Antoni S. Ferlay J. Soerjomataram I. Znaor A. Jemal A. Bray F. (2017). Bladder Cancer Incidence and Mortality: A Global Overview and Recent Trends. Eur. Urol. 71 (1), 96–108. 10.1016/j.eururo.2016.06.010 27370177

[B2] Boulay A. Masson R. Chenard M. P. El Fahime M. Cassard L. Bellocq J. P. (2001). High Cancer Cell Death in Syngeneic Tumors Developed in Host Mice Deficient for the Stromelysin-3 Matrix Metalloproteinase. Cancer Res. 61, 2189. 11280785

[B3] Cai L. Zhang Y. Zhang Y. Chen H. Hu J. (2019). Effect of Th9/IL-9 on the Growth of Gastric Cancer in Nude Mice. Ott 12, 2225–2234. 10.2147/ott.S197816 PMC6441462 30988627

[B4] Chamie K. Litwin M. S. Bassett J. C. Daskivich T. J. Lai J. Hanley J. M. (2013). Recurrence of High-Risk Bladder Cancer: a Population-Based Analysis. Cancer 119 (17), 3219–3227. 10.1002/cncr.28147 23737352 PMC3773281

[B5] Champelovier P. Boucard N. Levacher G. Simon A. Seigneurin D. Praloran V. (2002). Plasminogen- and colony-stimulating Factor-1-Associated Markers in Bladder Carcinoma: Diagnostic Value of Urokinase Plasminogen Activator Receptor and Plasminogen Activator Inhibitor Type-2 Using Immunocytochemical Analysis. Urol. Res. 30 (5), 301–309. 10.1007/s00240-002-0270-5 12389118

[B6] Chen L.-l. Zhang Z.-j. Yi Z.-b. Li J.-j. (2017). MicroRNA-211-5p Suppresses Tumour Cell Proliferation, Invasion, Migration and Metastasis in Triple-Negative Breast Cancer by Directly Targeting SETBP1. Br. J. Cancer 117 (1), 78–88. 10.1038/bjc.2017.150 28571042 PMC5520212

[B7] Chen N. Wang X. (2014). Role of IL-9 and STATs in Hematological Malignancies (Review). Oncol. Lett. 7 (3), 602–610. 10.3892/ol.2013.1761 24520283 PMC3919939

[B8] Chen W. Sun K. Sun K. Zheng R. Zeng H. Zhang S. (2018). Cancer Incidence and Mortality in China, 2014. Chin. J. Cancer Res. 30 (1), 1–12. 10.21147/j.issn.1000-9604.2018.01.01 29545714 PMC5842223

[B9] Couto P. Miranda D. Vieira R. Vilhena A. De Marco L. Bastos-Rodrigues L. (2014). Association between CLOCK, PER3 and CCRN4L with Non-small Cell Lung Cancer in Brazilian Patients. Mol. Med. Rep. 10 (1), 435–440. 10.3892/mmr.2014.2224 24821610

[B10] Englund E. Bartoschek M. Reitsma B. Jacobsson L. Escudero-Esparza A. Orimo A. (2016). Cartilage Oligomeric Matrix Protein Contributes to the Development and Metastasis of Breast Cancer. Oncogene 35 (43), 5585–5596. 10.1038/onc.2016.98 27065333

[B11] Farling K. B. (2017). Bladder Cancer: Risk Factors, Diagnosis, And Management. Nurse Pract. 42 (3), 26–33. 10.1097/01.NPR.0000512251.61454.5c 28169964

[B12] Ferlay J. Colombet M. Soerjomataram I. Mathers C. Parkin D. M. Piñeros M. (2019). Estimating the Global Cancer Incidence and Mortality in 2018: GLOBOCAN Sources and Methods. Int. J. Cancer 144 (8), 1941–1953. 10.1002/ijc.31937 30350310

[B13] Filipski E. Lévi F. (2009). Circadian Disruption in Experimental Cancer Processes. Integr. Cancer Ther. 8 (4), 298–302. 10.1177/1534735409352085 20042408

[B14] Ganesh S. Sier C. F. Griffioen G. Vloedgraven H. J. de Boer A. Welvaart K. (1994). Prognostic Relevance of Plasminogen Activators and Their Inhibitors in Colorectal Cancer. Cancer Res. 54 (15), 4065–4071. 8033138

[B15] Hautmann R. E. Gschwend J. E. de Petriconi R. C. Kron M. Volkmer B. G. (2006). Cystectomy for Transitional Cell Carcinoma of the Bladder: Results of a Surgery Only Series in the Neobladder Era. J. Urol. 176 (2), 486–492. 10.1016/j.juro.2006.03.038 16813874

[B16] Hedegaard J. Lamy P. Nordentoft I. Algaba F. Høyer S. Ulhøi B. P. (2016). Comprehensive Transcriptional Analysis of Early-Stage Urothelial Carcinoma. Cancer Cell 30 (1), 27–42. 10.1016/j.ccell.2016.05.004 27321955

[B17] Hu B. Qiu-Lan H. Lei R. E. Shi C. Jiang H. X. Qin S. Y. (2017). Interleukin-9 Promotes Pancreatic Cancer Cells Proliferation and Migration via the miR-200a/Beta-Catenin Axis. Biomed. Res. Int. 2017, 2831056. 10.1155/2017/2831056 28349057 PMC5352879

[B18] Kim M.-Y. Ann E.-J. Mo J.-S. Dajas-Bailador F. Seo M.-S. Hong J.-A. (2010). JIP1 Binding to RBP-Jk Mediates Cross-Talk between the Notch1 and JIP1-JNK Signaling Pathway. Cell Death Differ 17 (11), 1728–1738. 10.1038/cdd.2010.50 20508646

[B19] Kim W.-J. Bae S.-C. (2008). Molecular Biomarkers in Urothelial Bladder Cancer. Cancer Sci. 99 (4), 646–652. 10.1111/j.1349-7006.2008.00735.x 18377416 PMC11160052

[B20] Kim W.-J. Kim E.-J. Kim S.-K. Kim Y.-J. Ha Y.-S. Jeong P. (2010). Predictive Value of Progression-Related Gene Classifier in Primary Non-muscle Invasive Bladder Cancer. Mol. Cancer 9, 3. 10.1186/1476-4598-9-3 20059769 PMC2821358

[B21] Kim W.-J. Quan C. (2005). Genetic and Epigenetic Aspects of Bladder Cancer. J. Cel. Biochem. 95 (1), 24–33. 10.1002/jcb.20412 15759278

[B22] Kirkali Z. Chan T. Manoharan M. Algaba F. Busch C. Cheng L. (2005). Bladder Cancer: Epidemiology, Staging and Grading, and Diagnosis. Urology 66 (6), 4–34. 10.1016/j.urology.2005.07.062 16399414

[B23] Kong R. Feng J. Ma Y. Zhou B. Li S. Zhang W. (2016). Silencing NACK by siRNA Inhibits Tumorigenesis in Non-small Cell Lung Cancer via Targeting Notch1 Signaling Pathway. Oncol. Rep. 35 (4), 2306–2314. 10.3892/or.2016.4552 26782286

[B24] Lai J. P. Sandhu D. S. Shire A. M. Roberts L. R. (2008). The Tumor Suppressor Function of Human Sulfatase 1 (SULF1) in Carcinogenesis. J. Gastrointest. Cancer 39 (1-4), 149–158. 10.1007/s12029-009-9058-y 19373441 PMC2925118

[B25] Lee N. H. Park S. R. Lee J. W. Lim S. Lee S. H. Nam S. (2019). SERPINB2 Is a Novel Indicator of Cancer Stem Cell Tumorigenicity in Multiple Cancer Types. Cancers (Basel) 11 (4), 499. 10.3390/cancers11040499 PMC6520756 30965654

[B26] Li H. R. Gao J. Jin C. Jiang J. H. Ding J. Y. (2020). Downregulation of SETBP1 Promoted Non-small Cell Lung Cancer Progression by Inducing Cellular EMT and Disordered Immune Status. Am. J. Transl Res. 12 (2), 447–462. 32194895 PMC7061827

[B27] Liu F. Huang J. He F. Ma X. Fan F. Meng M. (2020). CD96, a New Immune Checkpoint, Correlates with Immune Profile and Clinical Outcome of Glioma. Sci. Rep. 10 (1), 10768. 10.1038/s41598-020-66806-z 32612110 PMC7330044

[B28] Liu T.-t. Liu X.-s. Zhang M. Liu X.-n. Zhu F.-x. Zhu F.-m. (2018). Cartilage Oligomeric Matrix Protein Is a Prognostic Factor and Biomarker of colon Cancer and Promotes Cell Proliferation by Activating the Akt Pathway. J. Cancer Res. Clin. Oncol. 144 (6), 1049–1063. 10.1007/s00432-018-2626-4 29560517 PMC11813401

[B29] Liu T. Han X. Zheng S. Liu Q. Tuerxun A. Zhang Q. (2021). CALM1 Promotes Progression and Dampens Chemosensitivity to EGFR Inhibitor in Esophageal Squamous Cell Carcinoma. Cancer Cel Int 21 (1), 121. 10.1186/s12935-021-01801-6 PMC7890995 33602237

[B30] Locasale J. W. Grassian A. R. Melman T. Lyssiotis C. A. Mattaini K. R. Bass A. J. (2011). Phosphoglycerate Dehydrogenase Diverts Glycolytic Flux and Contributes to Oncogenesis. Nat. Genet. 43 (9), 869–874. 10.1038/ng.890 21804546 PMC3677549

[B31] Meng J. Lu X. Zhou Y. Zhang M. Ge Q. Zhou J. (2021). Tumor Immune Microenvironment-Based Classifications of Bladder Cancer for Enhancing the Response Rate of Immunotherapy. Mol. Ther. - Oncolytics 20 (2021), 410–421. 10.1016/j.omto.2021.02.001 33665361 PMC7900642

[B32] Mo Q. Nikolos F. Chen F. Tramel Z. Lee Y.-C. Hayashi K. (2018). Prognostic Power of a Tumor Differentiation Gene Signature for Bladder Urothelial Carcinomas. JNCI: J. Natl. Cancer Inst. 110 (5), 448–459. 10.1093/jnci/djx243 29342309 PMC6279371

[B33] Pang C. Guan Y. Li H. Chen W. Zhu G. (2016). Urologic Cancer in China. Jpn. J. Clin. Oncol. 46 (6), 497–501. 10.1093/jjco/hyw034 27049022

[B34] Pontious C. Kaul S. Hong M. Hart P. A. Krishna S. G. Lara L. F. (2019). Cathepsin E Expression and Activity: Role in the Detection and Treatment of Pancreatic Cancer. Pancreatology 19 (7), 951–956. 10.1016/j.pan.2019.09.009 31582345 PMC6829043

[B35] Possemato R. Marks K. M. Shaul Y. D. Pacold M. E. Kim D. Birsoy K. (2011). Functional Genomics Reveal that the Serine Synthesis Pathway Is Essential in Breast Cancer. Nature 476 (7360), 346–350. 10.1038/nature10350 21760589 PMC3353325

[B36] Ross J. S. Fletcher J. A. Linette G. P. Stec J. Clark E. Ayers M. (2003). The Her-2/neu Gene and Protein in Breast Cancer 2003: Biomarker and Target of Therapy. Oncologist 8 (4), 307–325. 10.1634/theoncologist.8-4-307 12897328

[B37] Salazar Y. Zheng X. Brunn D. Raifer H. Picard F. Zhang Y. (2020). Microenvironmental Th9 and Th17 Lymphocytes Induce Metastatic Spreading in Lung Cancer. J. Clin. Invest. 130 (7), 3560–3575. 10.1172/JCI124037 32229721 PMC7324180

[B38] Sjödahl G. Lauss M. Lövgren K. Chebil G. Gudjonsson S. Veerla S. (2012). A Molecular Taxonomy for Urothelial Carcinoma. Clin. Cancer Res. 18 (12), 3377–3386. 10.1158/1078-0432.Ccr-12-0077-t 22553347

[B39] Song Z. Feng C. Lu Y. Lin Y. Dong C. (2018). PHGDH Is an Independent Prognosis Marker and Contributes Cell Proliferation, Migration and Invasion in Human Pancreatic Cancer. Gene 642, 43–50. 10.1016/j.gene.2017.11.014 29128633

[B40] Stanko K. Iwert C. Appelt C. Vogt K. Schumann J. Strunk F. J. (2018). CD96 Expression Determines the Inflammatory Potential of IL-9-producing Th9 Cells. Proc. Natl. Acad. Sci. USA 115 (13), E2940–e2949. 10.1073/pnas.1708329115 29531070 PMC5879650

[B41] Subramanian A. Tamayo P. Mootha V. K. Mukherjee S. Ebert B. L. Gillette M. A. (2005). Gene Set Enrichment Analysis: a Knowledge-Based Approach for Interpreting Genome-wide Expression Profiles. Proc. Natl. Acad. Sci. 102 (43), 15545–15550. 10.1073/pnas.0506580102 16199517 PMC1239896

[B42] Tian J. Lou J. Cai Y. Rao M. Lu Z. Zhu Y. (2020). Risk SNP-Mediated Enhancer-Promoter Interaction Drives Colorectal Cancer through Both FADS2 and AP002754.2. Cancer Res. 80 (9), 1804–1818. 10.1158/0008-5472.CAN-19-2389 32127356

[B43] Tsang J. Y. S. Tse G. M. (2020). Molecular Classification of Breast Cancer. Adv. Anat. Pathol. 27 (1), 27–35. 10.1097/pap.0000000000000232 31045583

[B44] Uno K. Azuma T. Nakajima M. Yasuda K. Hayakumo T. Mukai H. (2000). Clinical Significance of Cathepsin E in Pancreatic Juice in the Diagnosis of Pancreatic Ductal Adenocarcinoma. J. Gastroenterol. Hepatol. 15 (11), 1333–1338. 10.1046/j.1440-1746.2000.02351.x 11129230

[B45] Vriens K. Christen S. Parik S. Broekaert D. Yoshinaga K. Talebi A. (2019). Evidence for an Alternative Fatty Acid Desaturation Pathway Increasing Cancer Plasticity. Nature 566 (7744), 403–406. 10.1038/s41586-019-0904-1 30728499 PMC6390935

[B46] Weber S. Koschade S. E. Hoffmann C. M. Dubash T. D. Giessler K. M. Dieter S. M. (2019). The Notch Target Gene HEYL Modulates Metastasis Forming Capacity of Colorectal Cancer Patient-Derived Spheroid Cells *In Vivo* . BMC Cancer 19 (1), 1181. 10.1186/s12885-019-6396-4 31796022 PMC6892194

[B47] Yan D. Dai H. Liu J.-W. (2011). Serum Levels of MMP-11 Correlate with Clinical Outcome in Chinese Patients with Advanced Gastric Adenocarcinoma. BMC Cancer 11, 151. 10.1186/1471-2407-11-151 21513571 PMC3094327

[B48] Yu G. Wang L.-G. Han Y. He Q.-Y. (2012). clusterProfiler: an R Package for Comparing Biological Themes Among Gene Clusters. OMICS: A J. Integr. Biol. 16 (5), 284–287. 10.1089/omi.2011.0118 PMC3339379 22455463

[B49] Zhao X. Fu J. Du J. Xu W. (2020). The Role of D-3-Phosphoglycerate Dehydrogenase in Cancer. Int. J. Biol. Sci. 16 (9), 1495–1506. 10.7150/ijbs.41051 32226297 PMC7097917

[B50] Zhu S. Yu W. Yang X. Wu C. Cheng F. (2020). Traditional Classification and Novel Subtyping Systems for Bladder Cancer. Front. Oncol. 10, 102. 10.3389/fonc.2020.00102 32117752 PMC7025453

