# Supplementary material for: Construction and Validation of a 15-Top-prognostic-gene-based Signature to Indicate the Dichotomized Clinical Outcome and Response to Targeted Therapy for Bladder Cancer Patients
Source: Front Cell Dev Biol. 2022 Mar 31;10:725024. doi: 10.3389/fcell.2022.725024 (PMC9009041; doi:10.3389/fcell.2022.725024)
Supplement: Supplementary file 1 [file Image1.PDF]

Table S1. Sequences of negative control and HELY inhibitors.

siRNA	sequence
Negative control	5’-GGCUCUAGAAAAGCCUAUGC-3’
si-HEYL-1#	5’-GACGGUGGAUCACUUGAAA-3’
si-HEYL-2#	5’-CCACTGCCTTTGAGAAACA-3’

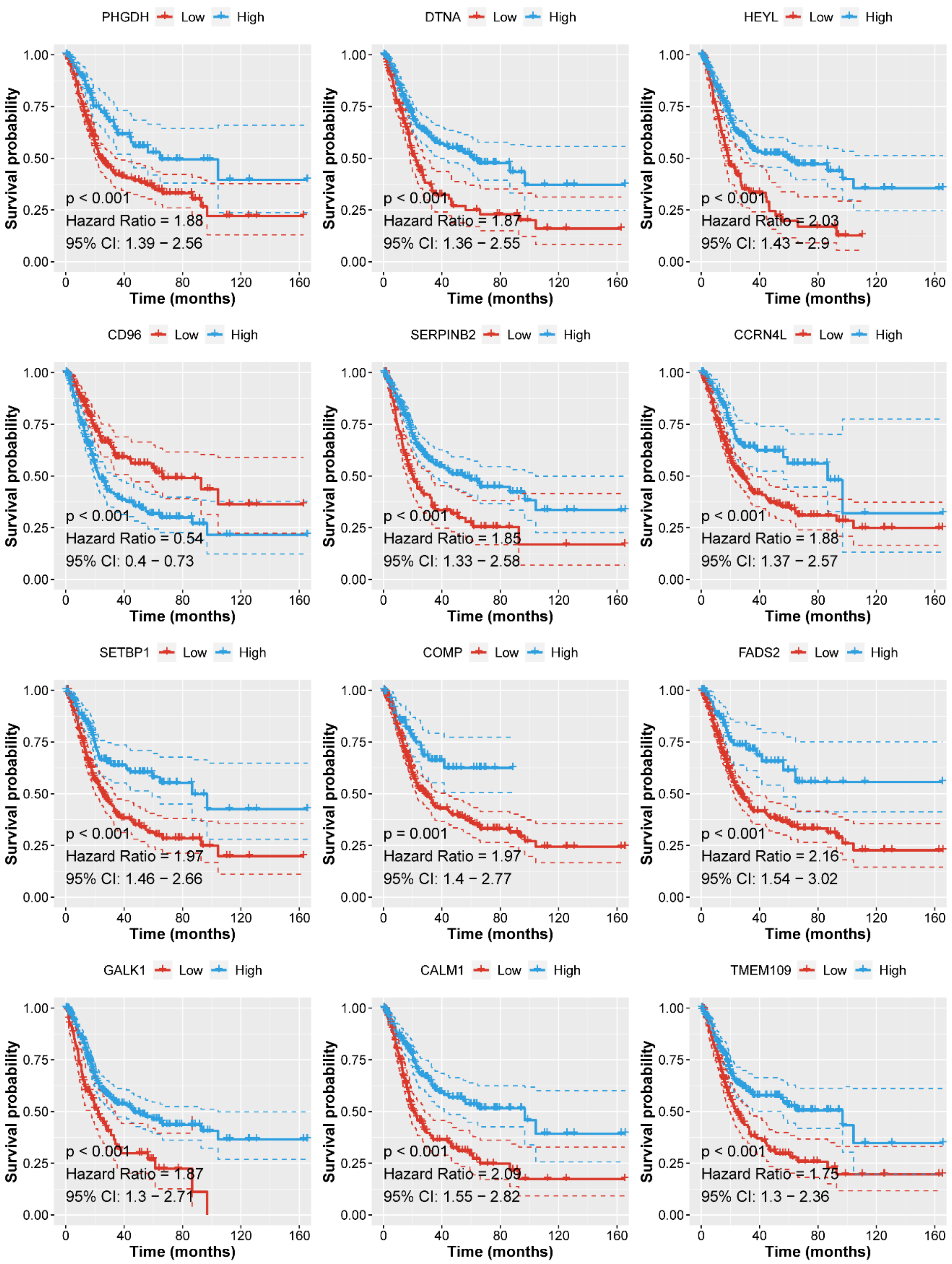
PHGDH Low High
p < 0.001
Hazard Ratio = 1.88
95% CI: 1.39 − 2.56
DTNA Low High
p < 0.001
Hazard Ratio = 1.87
95% CI: 1.36 − 2.55
HEYL Low High
p < 0.001
Hazard Ratio = 2.03
95% CI: 1.43 − 2.9
CD96 Low High
p < 0.001
Hazard Ratio = 0.54
95% CI: 0.4 − 0.73
SERPINB2 Low High
p < 0.001
Hazard Ratio = 1.85
95% CI: 1.33 − 2.58
CCRN4L Low High
p < 0.001
Hazard Ratio = 1.88
95% CI: 1.37 − 2.57
SETBP1 Low High
p < 0.001
Hazard Ratio = 1.97
95% CI: 1.46 − 2.66
COMP Low High
p = 0.001
Hazard Ratio = 1.97
95% CI: 1.4 − 2.77
FADS2 Low High
p < 0.001
Hazard Ratio = 2.16
95% CI: 1.54 − 3.02
GALK1 Low High
p < 0.001
Hazard Ratio = 1.87
95% CI: 1.3 − 2.71
CALM1 Low High
p < 0.001
Hazard Ratio = 2.09
95% CI: 1.55 − 2.82
TMEM109 Low High
p < 0.001
Hazard Ratio = 1.75
95% CI: 1.3 − 2.36
Survival probability
Time (months)
0.00
0.25
0.50
0.75
1.00
0
40
80
120
160

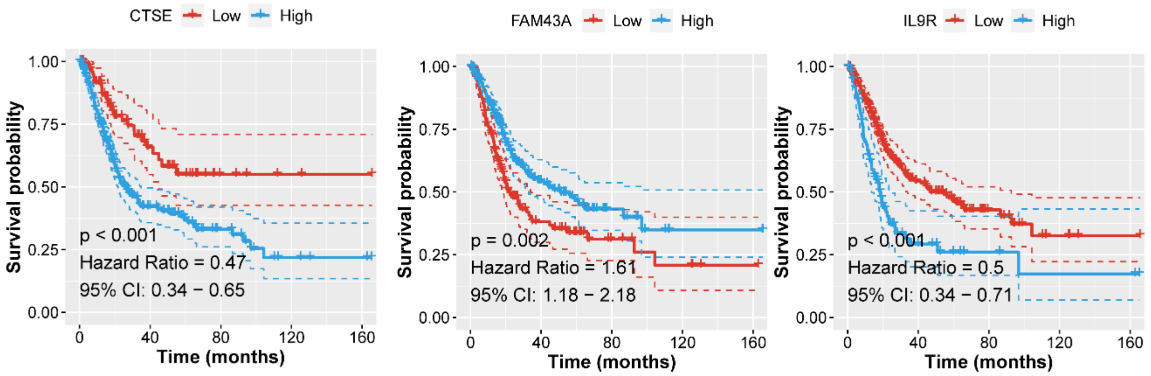

Figure S1. The prognostic value of enrolled 15 genes.

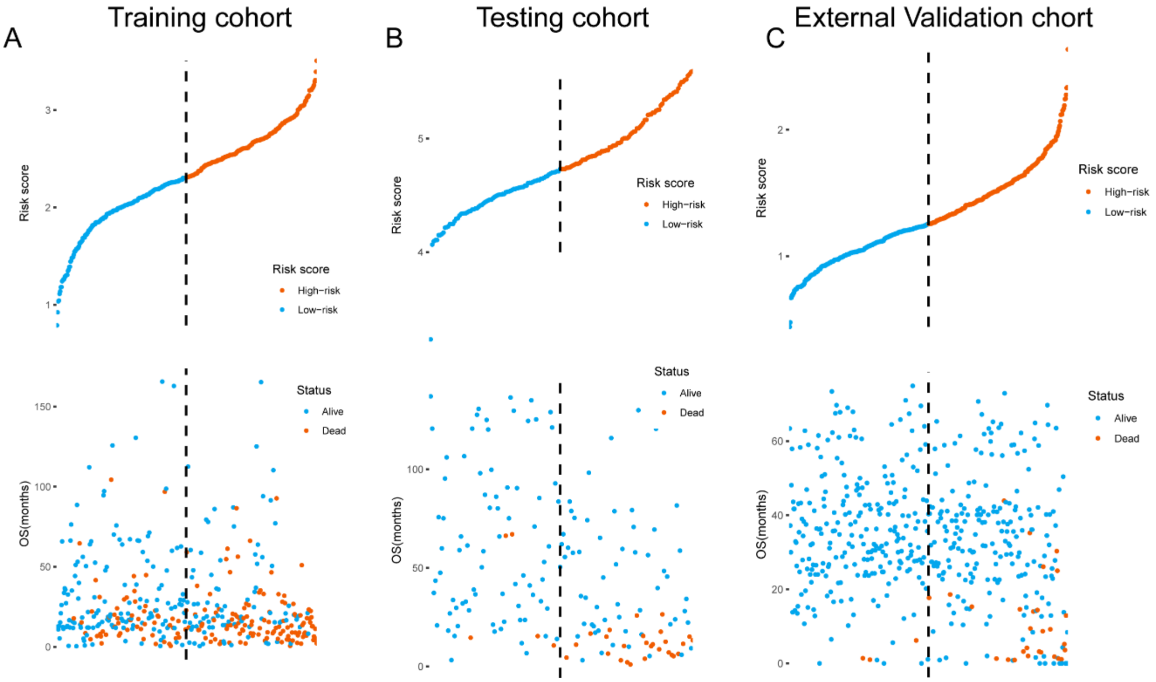

Figure S2. Distribution of risk score and survival status of patients. A. Training TCGA-BLCA cohort; B. Testing GSE13507 cohort; C. External validation E-MTAB4321 cohort.

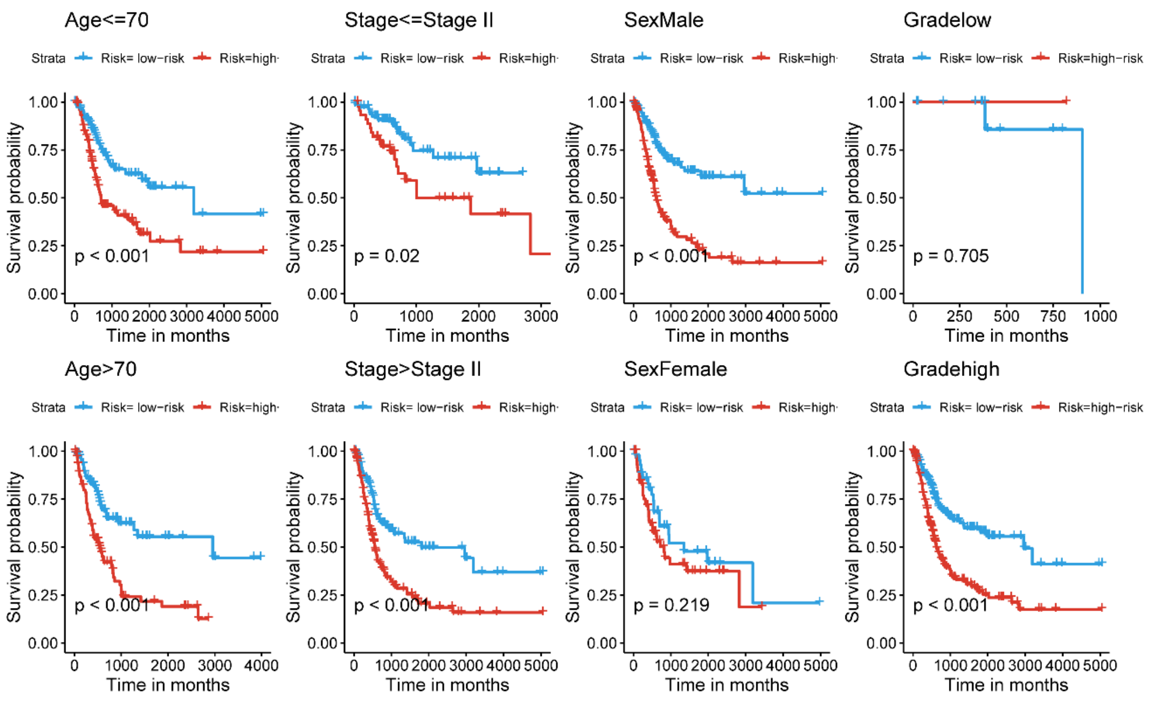

Figure S3. The prognostic value of the 15-gene signatures in the subgroup of TCGA-BLCA cohort.

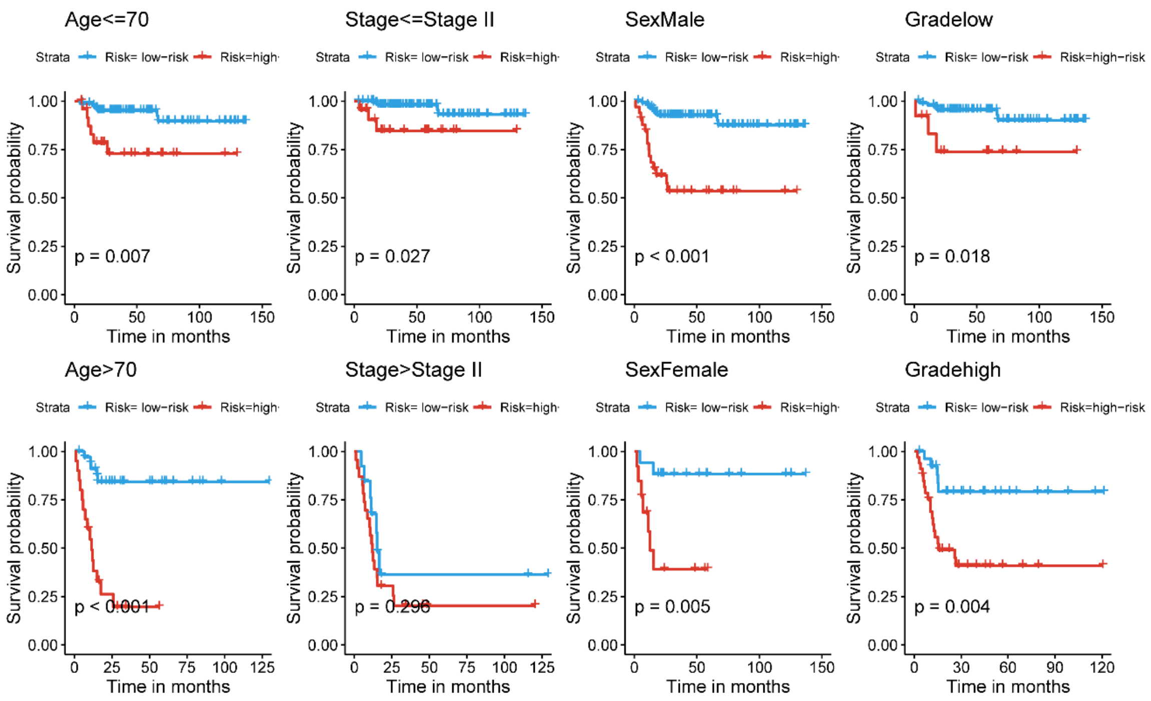

Figure S4. The prognostic value of the 15-gene signatures in the subgroup of GSE13507 cohort.

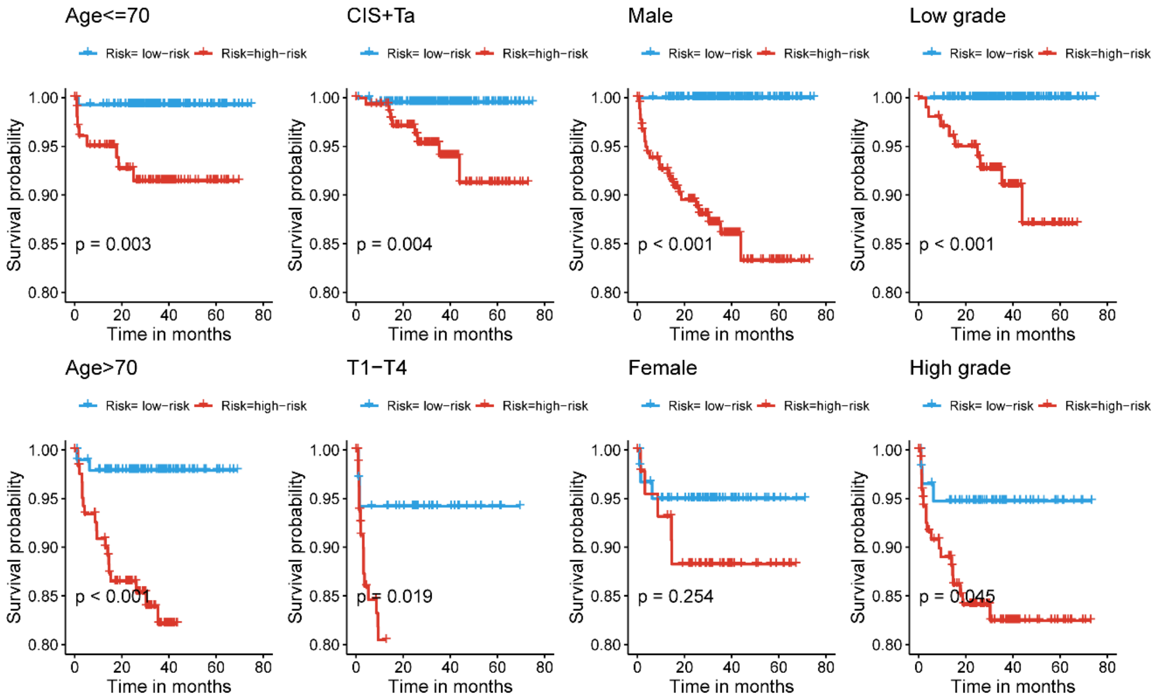

Figure S5. The prognostic value of the 15-gene signatures in the subgroup of E-MTAB-4321 cohort.
